# Joint UAV Placement and Active IRS Gain Optimization for Covert Communications

**DOI:** 10.3390/s26165244

**Published:** 2026-08-19

**Authors:** Guojie Qu, Mei Shen, Kai Liu, Bin Xu, Yuwen Qian

**Affiliations:** 1 College of Electrical Engineering, Sichuan University, Chengdu 610065, China; 2 FAW-Volkswagen Automotive Co., Ltd., Changchun 130011, China; 3 School of Electronic and Optical Engineering, Nanjing University of Science and Technology, Nanjing 210094, China; 4 College of Mechanical Engineering, Sichuan University, Chengdu 610065, China

**Keywords:** covert communications, intelligent reflecting surface, unmanned aerial vehicle, Kullback–Leibler divergence, joint optimization

## Abstract

Wireless sensing networks increasingly extend into obstacle-prone deployments, where physical blockage degrades reliability and open propagation exposes transmission activity. Intelligent reflecting surfaces (IRSs) establish programmable paths around obstacles while passive elements remain constrained by severe cascaded attenuation. To address the tradeoff between reliability and covertness, we propose an unmanned aerial vehicle (UAV) -assisted active-IRS architecture under probabilistic line-of-sight and non-line-of-sight propagation conditions that accounts for direct leakage from the transmitter to the warden together with residual jammer cancellation and always-on IRS circuit noise under a finite output power budget. Furthermore, bidirectional Kullback–Leibler analysis identifies the reverse divergence as the tighter restriction and converts the covertness requirement into conservative gain bounds under warden location uncertainty and relative phase uncertainty conditions between the direct and aggregate reflected fields. Subsequently, closed-form phase control for calibrated equal-gain elements and gain monotonicity reduce the joint design to an exhaustive search over the prescribed placement grid. The numerical results demonstrate a SINR advantage over passive reflection and single-element relaying across the evaluated settings. The finite-array and hardware analyses show that gain back-off enforces a prescribed covert-outage limit while direct leakage and residual self-interference remain explicitly controlled. Overall, the framework provides a transparent basis for reliable covert sensing through UAV-assisted active reflection.

## 1. Introduction

Unmanned aerial vehicles (UAVs) provide rapid three-dimensional connectivity for long-distance sensing networks under obstacle-prone propagation conditions. Flexible placement improves air-to-ground visibility and bypasses fixed terrestrial blockage. Wireless sensing traffic often contains private measurements that require reliable delivery and low detectability. Encryption protects message content while physical-layer security exploits channel asymmetry. Neither mechanism hides transmission activity from an observer. Covert communication instead limits the statistical evidence available for signal-presence detection. Unlike secrecy, covertness seeks to conceal the occurrence of transmission rather than only its content. In standard covert communication terminology, Alice denotes the covert transmitter, while Bob denotes the intended receiver, and Willie denotes the passive warden that tests whether Alice is active. A higher source power improves a blocked link while it strengthens the evidence observed by Willie. Aerial geometry reduces path loss while an active reflecting surface supplies controllable amplification around obstacles. The central design problem is therefore to balance reliable reception against a quantitative detection limit.

While covert waveform design reduces detection evidence through adversarial learning and chaotic modulation [1], conventional relays extend coverage around blockage through an additional forwarding stage. Rate-adaptive multicast extends wireless coverage through coordinated channel and transmission-rate selection [2]. Channel uncertainty at a trusted relay supports covert transmission in a practical radiometer model [3]. In heterogeneous Internet of Things systems, relay selection improves the covert rate relative to random selection when candidate links have different qualities [4]. Full-duplex operation subsequently coordinates relay mode and power under different forwarding conditions [5]. Energy harvesting further sustains hidden transmission when relay power depends on the received source signal [6]. Multiple antennas then support directional forwarding under optimized beamforming conditions [7]. A cooperative relay injects artificial noise in addition to forwarding the confidential signal. Its receiver and transmitter introduce processing noise together with radio-frequency power demand. Rate adaptation changes the operating point while the relay geometry remains fixed. An unfavorable warden location therefore continues to restrict the forwarding path. Moreover, a conventional relay must estimate the incoming waveform before amplification or decoding. Every additional operation increases the burden on an aerial platform with limited energy and payload. Consequently, conventional relaying provides useful rate and forwarding control while its fixed geometry preserves a placement-dependent detection problem. A lower-complexity aerial architecture with flexible spatial control therefore remains important for blocked covert links.

However, UAV platforms turn relay placement into an adaptable design variable through altitude and horizontal motion. Flexible deployment and favorable air-to-ground channels support temporary coverage [8]. Coordination between aerial and ground resources within the low-altitude economy further supports adaptive sensing and communication services [9]. Successive placement algorithms reduce the number of UAV-mounted base stations required for ground coverage [10]. Resource-aware transition planning preserves multihop multicast service during UAV motion [11]. Coverage and transition methods optimize service objectives rather than covert detection. For covert data collection, random artificial noise from a full-duplex UAV protects transmissions from ground devices [12]. In random warden locations, a multi-antenna jammer directs interference through an outage formulation [13]. Trajectory and transmit-power optimization then improves the average covert rate when a UAV acts as a mobile transmitter [14]. Within relay networks, placement optimization increases effective throughput under packet-length and power constraints [15]. Joint trajectory and resource allocation further extends UAV relaying to sensor association under noise uncertainty conditions [16]. Collectively, prior formulations establish the value of aerial mobility for communication and detection control. Most formulations employ the UAV as a transmitter or receiver. Other designs treat the UAV as a jammer or a conventional active relay. Conventional aerial relaying performs signal reception and baseband processing before retransmission. Its active transceiver consumes onboard power throughout the forwarding interval. Moreover, favorable line-of-sight propagation exposes the transmitted waveform to a ground warden. Mixed line-of-sight and non-line-of-sight modeling is therefore required when urban blockage changes with elevation and distance. A lightweight aerial architecture must therefore retain geometric control without the complete transceiver burden of conventional relaying.

Nevertheless, an intelligent reflecting surface (IRS) replaces a relay receiver chain with element-wise propagation control and limited radio-frequency processing. The foundational formulation for IRS resource allocation jointly optimizes transmit and reflection beamforming [17]. Early covert IRS architectures considered blockage support and interference control in several operating modes [18]. With statistical channel information at Willie, joint source-power and reflection design has been formulated with an outage objective [19]. Delay-constrained short-packet transmission subsequently links IRS configuration with finite communication time [20]. Multi-antenna formulations further coordinate transmit beamforming with IRS reflection under a covertness constraint [21]. From a detection perspective, the relative IRS distances govern whether reflection benefits Bob or Willie [22]. A two-stage method separates reflection beamforming from covert power allocation to reduce computational cost [23]. IRS-assisted nonorthogonal multiple access then couples public and covert users within one reflection design [24]. Related physical-layer security designs coordinate reconfigurable intelligent surface (RIS) phase control and artificial noise for full-duplex integrated sensing and communication under secrecy constraints [25]. Wireless-powered hybrid surfaces coordinate three element modes with amplification and resource allocation [26]. Conventional and hybrid RIS formulations optimize beamforming or operating modes without aerial placement. Passive reflection requires little radio-frequency power and avoids relay decoding. Each reflected component nonetheless traverses the Alice-to-IRS link and the IRS-to-Bob link. The cascaded channel gain therefore contains the product of both path gains. Phase alignment recovers an $M^{2}$ array factor at Bob but does not remove severe double attenuation over a long route. Active reflection counters the cascaded attenuation through a controllable gain while it introduces circuit noise and a finite output budget.

Although UAV-enabled IRSs combine geometric freedom with programmable propagation, current covert formulations largely retain passive reflection. Joint UAV trajectory and phase optimization improves covert performance with a movable aerial surface [27]. Random IRS phases then introduce additional uncertainty into Willie’s observation model [28]. Related UAV–RIS research further accounts for platform jitter and hardware impairment through joint beamforming and trajectory control [29]. Energy-efficiency optimization extends covert coverage through an aerial reconfigurable surface [30]. Age-of-information design further applies an aerial IRS to timely covert data collection in Internet of Things networks [31]. Prior aerial IRS formulations therefore emphasize passive phase control and trajectory planning. Their reliability benefit remains tied to a unit-modulus surface under cascaded attenuation conditions. Active reflection supplies additional link gain but changes the forwarded-noise model and the aerial power constraint. Friendly jamming changes Willie’s null-hypothesis variance and further couples placement with reflection gain. A complete design must therefore connect the aerial geometry to Willie’s detector before the active gain is selected. Existing work rarely combines mixed line-of-sight propagation with friendly aerial jamming within one placement model. Few formulations integrate direct Willie leakage and active-IRS circuit noise with a finite output budget. The missing connection obscures which physical constraint limits amplification at each candidate location. The present methodology begins with Willie’s detector rather than a direct joint search over geometry and surface coefficients. Kullback–Leibler (KL) inversion produces a gain ceiling at each location before closed-form phase alignment removes the element phases from the placement search. The primary methodological novelty is the resulting sequence from detector inversion to phase elimination and constrained spatial optimization. Accordingly, three coupled research problems remain. The observation model must retain direct leakage and residual hardware interference. The covertness restriction must remain tractable under warden location uncertainty and finite-array fading conditions. IRS phase and active gain must then be coordinated with UAV placement under a finite output budget.

To address these issues, we developed a UAV-enabled active-IRS framework for a blocked covert link. The proposed model connects Willie’s binary detector with Bob’s reflected channel and separates pointwise gain selection from the remaining placement search. The main contributions are summarized as follows.

- We propose a UAV-enabled active-IRS covert communication framework in which the aerial platform supplies friendly jamming and controlled reflection for a blocked Alice-to-Bob link under probabilistic line-of-sight (LoS) and non-line-of-sight (NLoS) propagation conditions. The framework retains direct Alice-to-Willie leakage and residual jammer interference while it accounts for IRS circuit noise and a finite output-power budget.
- To characterize Willie’s detection performance, we derive the forward and reverse Kullback–Leibler divergences and prove that the reverse divergence imposes the tighter restriction for every nonnegative observation signal-to-noise ratio (SNR). Accordingly, the covertness requirement yields a conservative gain bound in uncertain Willie locations and relative phase uncertainty between the direct and aggregate reflected fields while the stated Rayleigh leakage model establishes a finite-array safety factor under a covert-outage limit.
- We formulated a joint UAV placement and active-IRS power gain problem and solved the calibrated equal-gain phase subproblem in closed form at every candidate location. The gain monotonicity then yields a pointwise solution while a two-dimensional procedure returns the exact finite-grid maximizer for a nominal Willie location and a finite-grid design with certified covertness under bounded location uncertainty conditions.

The remainder of the paper is organized as follows. Section 2 establishes the air-to-ground channels and binary observation model. Section 3 converts Willie’s detection requirement into a gain bound. Section 4 derives the pointwise optimal gain and the UAV placement procedure. Section 5 evaluates the proposed design against benchmark architectures. Section 6 summarizes the main findings.

## 2. System Model

**Figure 1:**
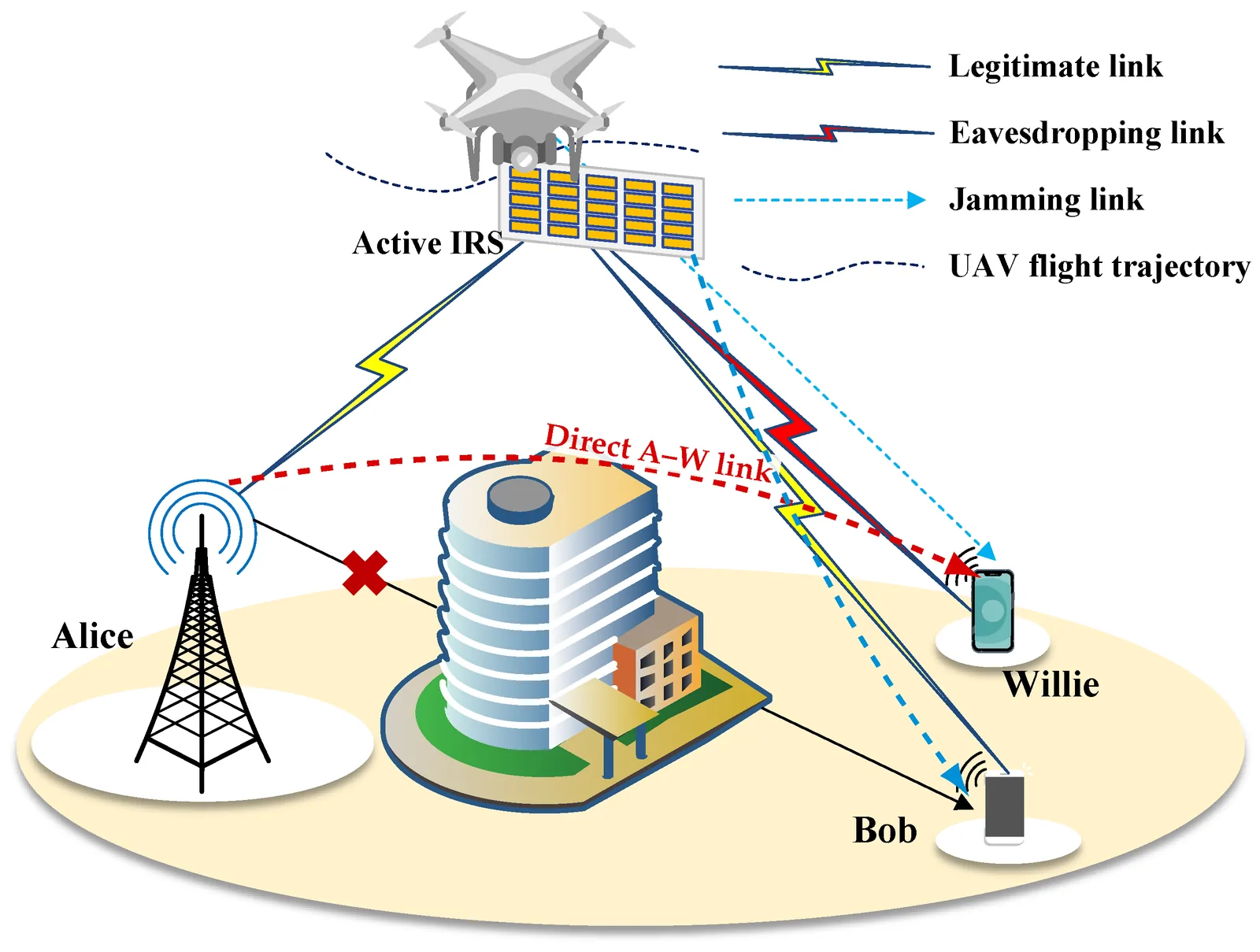
Covert communication architecture with an active intelligent reflecting surface (IRS) carried by an unmanned aerial vehicle (UAV) under direct Alice-to-Willie leakage and jammer coupling to both receivers.

As illustrated in Figure 1, Alice transmits confidential symbols to Bob through an active IRS carried by an UAV while Willie monitors the channel for transmission activity. Severe blockage eliminates direct Alice-to-Bob propagation, whereas the direct Alice-to-Willie link remains available. To mask Alice’s activity, a friendly jammer colocated with the IRS emits a seeded Gaussian waveform. Bob obtains the seed over a protected control link and estimates the jammer-to-Bob channel from pilots before waveform cancellation. The residual cancellation error is represented by a power ratio without invoking spatial nulling.

To parameterize the geometry in Figure 1, let $q_{k}={\left[x_{k},y_{k},0\right]}^{T}$ denote the single-antenna location of node k∈K≜{A,B,W}. The UAV is located at $q={\left[x,y,H\right]}^{T}$ where its horizontal projection $q_{h}={\left[x,y\right]}^{T}$ lies in $Q=\left[x_{\min},x_{\max}\right]\times \left[y_{\min},y_{\max}\right]$. At each feasible position, the UAV carries an active IRS with *M* elements and a common adjustable power gain.

Since placement uses Willie’s estimated position and large-scale channel statistics, the horizontal uncertainty is represented by

(1) $$U_{W}=\left\{q_{W}:{∥q_{W,h}-{\hat{q}}_{W,h}∥}_{2}\leq r_{W}\right\},$$

where ${\hat{q}}_{W,h}$ denotes the nominal horizontal location and $r_{W}$ is the uncertainty radius. A protected-zone boundary or prior radio map specifies $U_{W}$ without requiring signaling from Willie. The singleton case $r_{W}=0$ recovers the nominal-location baseline, whereas $r_{W}>0$ requires covertness throughout $U_{W}$.

### 2.1. Air-to-Ground Channel Model

At UAV location q, the distance and elevation angle to node *k* are

(2) $$d_{k}\left(q\right)={∥q-q_{k}∥}_{2},\;\vartheta_{k}\left(q\right)=\frac{180}{\pi }\arcsin\;\left(\frac{H}{d_{k}\left(q\right)}\right).$$

The elevation angle controls the LoS probability through

(3) $$p_{k}^{L}\left(q\right)=\frac{1}{1+a\exp\;\left[-b\left(\vartheta_{k}\left(q\right)-a\right)\right]},$$

where *a* and *b* describe the propagation environment. The LoS probability weights the normalized LoS and NLoS losses to form

(4) $$L_{k}\left(q\right)=p_{k}^{L}\left(q\right){\left[\frac{d_{k}\left(q\right)}{d_{0}}\right]}^{\alpha_{L}}+\left[1-p_{k}^{L}\left(q\right)\right]{\left[\frac{d_{k}\left(q\right)}{d_{0}}\right]}^{\alpha_{\mathrm{NL}}},$$

where $\alpha_{L}$ and $\alpha_{\mathrm{NL}}$ denote the LoS and NLoS exponents while $d_{0}=1$ m is the reference distance. With reference gain $\beta_{0}$ at 1 m, the large-scale channel power gain becomes

(5) $$G_{k}\left(q\right)=\frac{\beta_{0}}{L_{k}\left(q\right)},$$

To preserve Willie’s uncertain position, we write $G_{W}\left(q;q_{W}\right)$ for every $q_{W}\in U_{W}$. Under the co-location and equal-pattern assumptions, the jammer and IRS share the same large-scale gain toward each ground node. Deterministic large-scale gains and geometric element phases define the nominal channel vectors from Alice to the IRS and from the IRS to k∈{B,W} as

(6) $$h_{A}=\sqrt{G_{A}}{\left[e^{j\psi_{A,1}},\ldots ,e^{j\psi_{A,M}}\right]}^{T},\;h_{k}=\sqrt{G_{k}}{\left[e^{j\psi_{k,1}},\ldots ,e^{j\psi_{k,M}}\right]}^{T}.$$

The nominal vectors define a deterministic-equivalent baseline. Finite-array departures are represented by independent variables $g_{A,m},g_{W,m}∼\mathrm{CN}\left(0,1\right)$ that multiply the Alice-to-element and element-to-Willie coefficients. Each multiplier remains constant across the *n* detector samples and varies independently between coherence blocks. Section 3 uses the resulting leakage distribution to impose a covert-outage probability. Since the aerial model excludes direct terrestrial leakage, the Alice-to-Willie channel is modeled as

(7) $$h_{AW}\left(q_{W}\right)=\sqrt{G_{AW}\left(q_{W}\right)}e^{j\psi_{AW}},\;G_{AW}\left(q_{W}\right)=10^{-L_{AW}\left(q_{W}\right)/10},$$

where $L_{AW}\left(q_{W}\right)$ is the site-specific terrestrial path loss in dB. Blockage of the Alice-to-Bob path therefore does not imply blockage of the Alice-to-Willie path. Over each subregion of $U_{W}$, the terrestrial model supplies lower and upper bounds on $L_{AW}$. Equation (7) maps those bounds into certified interval enclosures of $G_{AW}$.

### 2.2. Active IRS Transmission Model

To mask Alice’s activity, the friendly jammer transmits z[i]∼CN(0,1) with power $P_{J}$. Bob reconstructs the seeded waveform through the protected control link and cancels it before decoding. The residual jamming-power ratio at Bob is $\rho_{B}\in \left[0,1\right]$ and corresponds to the cancellation level $C_{B}=-10\log_{10}\rho_{B}$ dB. Jammer leakage reaches the active IRS front end despite physical separation and absorptive shielding. Analog cancellation suppresses the known component before the active element amplifiers. After both suppression stages, $\eta_{\mathrm{SI}}$ denotes the residual power-coupling ratio at each element. The coupled leakage and circuit noise form spatially white distortion that remains uncorrelated with z[i]. Its per-element variance is

(8) $$\nu_{U}=\sigma_{U}^{2}+\eta_{\mathrm{SI}}P_{J},$$

where $\sigma_{U}^{2}$ denotes the circuit-noise variance. Consequently, the effective IRS noise satisfies ${\tilde{n}}_{U}∼\mathrm{CN}\left(0,\nu_{U}I_{M}\right)$, and the reflection matrix takes the form

(9) $$\Theta =\sqrt{\beta }\mathrm{diag}\;\left(e^{j\phi_{1}},\ldots ,e^{j\phi_{M}}\right),$$

where β≥0 is the common power gain. Alice transmits s[i]∼CN(0,1) with power $P_{A}$, while ${\tilde{z}}_{B}\left[i\right]∼\mathrm{CN}\left(0,1\right)$ denotes the normalized residual jammer waveform after cancellation. Bob therefore observes

(10) $$y_{B}\left[i\right]=\sqrt{P_{A}}h_{B}^{H}\Theta h_{A}s\left[i\right]+\sqrt{\rho_{B}P_{J}}h_{UB}{\tilde{z}}_{B}\left[i\right]+h_{B}^{H}\Theta {\tilde{n}}_{U}+n_{B}\left[i\right],$$

where $|h_{UB}{|}^{2}=G_{B}$ and $n_{B}\left[i\right]∼\mathrm{CN}\left(0,\sigma_{B}^{2}\right)$. At each UAV candidate location, closed-form phase control maximizes the desired term according to

(11) $$\begin{array}{cc} \phi^{★}\left(q\right)\; & \in \underset{0\leq \phi_{m}<2\pi }{\mathrm{arg max}}\;{\left|h_{B}^{H}\mathrm{diag}\;\left(e^{j\phi_{1}},\ldots ,e^{j\phi_{M}}\right)h_{A}\right|}^{2},\\ \phi_{m}^{★}\left(q\right) & =\psi_{B,m}\left(q\right)-\psi_{A,m}\left(q\right),\;m\in \left\{1,\ldots ,M\right\}. \end{array}$$

For calibrated equal-gain elements with spatially white distortion, Equation (11) is the global solution of the fixed-location phase subproblem. The phase vector is therefore updated before gain and placement are evaluated at every grid point. Willie’s noncoherent leakage depends on the reflected power sum and requires no instantaneous Willie CSI in the phase subproblem. After phase alignment, Bob’s deterministic-equivalent signal-to-interference-plus-noise ratio (SINR) becomes

(12) $$\gamma_{B}\left(q,\beta \right)=\frac{P_{A}M^{2}\beta G_{A}\left(q\right)G_{B}\left(q\right)}{M\beta \nu_{U}G_{B}\left(q\right)+\rho_{B}P_{J}G_{B}\left(q\right)+\sigma_{B}^{2}}.$$

Equation (12) reveals an $M^{2}$ coherent signal gain against an *M*-order forwarded-distortion term. Residual waveform cancellation contributes $\rho_{B}P_{J}G_{B}$ to the denominator.

UAV vibration and attitude variation introduce differential phase errors. Let the implemented phase be $\phi_{m}^{★}+\delta_{m}$ where the independent error satisfies $\delta_{m}∼N\left(0,\sigma_{\phi }^{2}\right)$. In this model, the expected coherent array factor is

(13) $$E\;\left[{\left|\sum_{m=1}^{M}e^{j\delta_{m}}\right|}^{2}\right]=M+M\left(M-1\right)e^{-\sigma_{\phi }^{2}}.$$

Equation (13) characterizes independent jitter. A correlated model instead sets $\delta ∼N(0,\Sigma_{\phi })$ and assigns a normalized measured amplitude response $r_{m}\left(\phi \right)\in \left[0,1\right]$ to element *m*. Its reflection matrix and expected array factor are

(14) $$\begin{array}{cc} \chi_{m}\left(\phi_{m}\right) & =\psi_{A,m}-\psi_{B,m}+\phi_{m},\\ \Theta_{\mathrm{hw}}\left(\phi ,\delta \right)\; & =\sqrt{\beta }\mathrm{diag}\;{\left(r_{m}\left(\phi_{m}+\delta_{m}\right)e^{j(\phi_{m}+\delta_{m})}\right)}_{m=1}^{M},\\ A_{\mathrm{hw}}\left(\phi \right)\; & =\sum_{m=1}^{M}\sum_{l=1}^{M}E\;\left[r_{m}\left(\phi_{m}+\delta_{m}\right)r_{l}\left(\phi_{l}+\delta_{l}\right)e^{j[\chi_{m}\left(\phi_{m}\right)-\chi_{l}\left(\phi_{l}\right)+\delta_{m}-\delta_{l}]}\right],\\ A_{\phi }\; & ={\left.A_{\mathrm{hw}}\left(\phi^{★}\right)\right|}_{r_{m}\left(\cdot \right)=1}=\sum_{m=1}^{M}\sum_{l=1}^{M}\exp\;\left[-\frac{1}{2}\mathrm{Var}\left(\delta_{m}-\delta_{l}\right)\right]. \end{array}$$

With unit phase-invariant amplitude, a common phase rotation preserves array power, whereas differential errors reduce $A_{\phi }$. For $r_{m}\left(\phi \right)=1$, $A_{\mathrm{hw}}\left(\phi^{★}\right)=A_{\phi }$. The calibrated baseline further sets $\delta_{m}=0$ and yields $A_{\mathrm{hw}}\left(\phi^{★}\right)=A_{\phi }=M^{2}$. Thus, Equation (11) is globally optimal only with the calibrated response. A measured response table enters the phase subproblem through $r_{m}\left(\phi_{m}\right)$ and requires numerical phase optimization at each location.

Once $A_{\mathrm{hw}}$ is fixed, the gain monotonicity in Equation (41) and the placement reduction remain unchanged. For a specified response, $A_{\mathrm{hw}}$ replaces $M^{2}$ in Equation (12). The retained $M\beta \nu_{U}G_{B}$ term upper-bounds forwarded distortion and yields a conservative SINR lower bound under $\sum_{m}r_{m}^{2}\left(\phi_{m}\right)\leq M$. The same inequality bounds signal leakage at Willie and the IRS output power.

Within each coherence interval, inertial sensing and repeated pilots support attitude compensation. The shared commanded gain is represented by β, whereas $r_{m}\left(\phi \right)$ describes the realized response. A finite output budget limits the aggregate radio-frequency power across all elements. Under hardware impairment conditions, joint UAV and IRS design determines the update rate and phase margin [29].

### 2.3. Willie Observation Model

Willie applies a binary hypothesis test to *n* samples collected within one coherence block. Let $h_{UW}$ denote the UAV-to-Willie jammer channel with $|h_{UW}{|}^{2}=G_{W}$. Since the jammer and active IRS use identical settings under both hypotheses, Willie’s samples follow

(15) $$y_{W}\left[i\right]=\left\{\begin{array}{cc} \sqrt{P_{J}}h_{UW}z\left[i\right]+h_{W}^{H}\Theta {\tilde{n}}_{U}\left[i\right]+n_{W}\left[i\right], & H_{0},\\ \sqrt{P_{A}}\;\left(h_{AW}+h_{W}^{H}\Theta h_{A}\right)s\left[i\right]+\sqrt{P_{J}}h_{UW}z\left[i\right]+h_{W}^{H}\Theta {\tilde{n}}_{U}\left[i\right]+n_{W}\left[i\right], & H_{1}, \end{array}\right.$$

where $n_{W}\left[i\right]∼\mathrm{CN}\left(0,\sigma_{W}^{2}\right)$. Identical IRS operation places the same amplified distortion under $H_{0}$ and $H_{1}$.

Since Equation (11) targets Bob, the reflected components retain residual phases at Willie. Let $\theta_{m}=\psi_{A,m}-\psi_{W,m}+\phi_{m}^{★}-\psi_{AW}$ denote the phase of element *m* relative to the direct field. The noncoherent model treats ${\left\{\theta_{m}\right\}}_{m=1}^{M}$ as independent uniform variables on [0,2π). Phase independence removes the expected cross terms and yields the leakage power

(16) $$E\;\left[{\left|h_{AW}+h_{W}^{H}\Theta h_{A}\right|}^{2}\right]=G_{AW}\left(q_{W}\right)+M\beta G_{A}\left(q\right)G_{W}\left(q;q_{W}\right).$$

The second moment in Equation (16) defines an equivalent Gaussian detector model that admits the exact KL expressions in Section 3. Its variances under $H_{0}$ and $H_{1}$ are

(17) $$\begin{array}{cc} \lambda_{0} & =P_{J}G_{W}\left(q;q_{W}\right)+M\beta \nu_{U}G_{W}\left(q;q_{W}\right)+\sigma_{W}^{2},\\ \lambda_{1} & =\lambda_{0}+P_{A}\;\left[G_{AW}\left(q_{W}\right)+M\beta G_{A}\left(q\right)G_{W}\left(q;q_{W}\right)\right]. \end{array}$$

The incremental signal variance yields Willie’s effective observation SNR

(18) $$\gamma_{W}\left(q,\beta ;q_{W}\right)=\frac{P_{A}\;\left[G_{AW}\left(q_{W}\right)+M\beta G_{A}\left(q\right)G_{W}\left(q;q_{W}\right)\right]}{P_{J}G_{W}\left(q;q_{W}\right)+M\beta \nu_{U}G_{W}\left(q;q_{W}\right)+\sigma_{W}^{2}}.$$

IRS distortion contributes equally under both hypotheses and raises their common variance. Its exclusion from Willie’s denominator produces the conservative upper bound

(19) $$\gamma_{W}\left(q,\beta ;q_{W}\right)\leq \gamma_{W}^{c}\left(q,\beta ;q_{W}\right)≜\frac{P_{A}\;\left[G_{AW}\left(q_{W}\right)+M\beta G_{A}\left(q\right)G_{W}\left(q;q_{W}\right)\right]}{P_{J}G_{W}\left(q;q_{W}\right)+\sigma_{W}^{2}}.$$

Placement optimization therefore enforces $\gamma_{W}^{c}$, whereas the circuit-noise analysis evaluates the exact divergence through $\gamma_{W}$. Equations (12) and (19) preserve the coherent $M^{2}$ gain toward Bob and the noncoherent *M*-order leakage toward Willie. The robust detector constraint takes the supremum over $U_{W}$ without instantaneous Willie phase information. Section 3 further quantifies block-level departures through a finite-array chance constraint.

## 3. Covertness Characterization via KL Divergence

Willie’s observation statistics permit a scalar reformulation of the covertness requirement. Let $P_{0}^{n}$ and $P_{1}^{n}$ denote the joint observation distributions under $H_{0}$ and $H_{1}$. Willie’s total detection error is $\xi =P_{\mathrm{FA}}+P_{\mathrm{MD}}$ [32]. Pinsker’s inequality yields

(20) $$\xi \geq 1-\sqrt{\frac{1}{2}D\;\left(P_{0}^{n}∥P_{1}^{n}\right)}.$$

Total variation is symmetric and therefore admits a Pinsker bound from either KL direction. We impose both directions and retain the stricter restriction. Hence, the target ξ≥1−ϵ is enforced by

(21) $$D\;\left(P_{0}^{n}∥P_{1}^{n}\right)\leq 2\epsilon^{2},\;D\;\left(P_{1}^{n}∥P_{0}^{n}\right)\leq 2\epsilon^{2}.$$

With the nominal equivalent power model, each sample follows a zero-mean circularly symmetric complex Gaussian distribution. Within a quasi-static fading block, the same density applies conditionally after $\lambda_{1}$ is replaced by its block value. The density under $H_{l}$ is

(22) $$p_{l}\left(y\right)=\frac{1}{\pi \lambda_{l}}\exp\;\left(-\frac{{\left|y\right|}^{2}}{\lambda_{l}}\right),\;l\in \left\{0,1\right\}.$$

Independence across the *n* samples makes each joint divergence *n* times its single-sample value. Substitution of Equations (17) and (18) yields

(23) $$\begin{array}{cc} D_{01} & =D\;\left(P_{0}^{n}∥P_{1}^{n}\right)=nE_{0}\;\left[\ln\frac{p_{0}\left(y\right)}{p_{1}\left(y\right)}\right]\\ \; & =n\left[\ln\frac{\lambda_{1}}{\lambda_{0}}+\frac{\lambda_{0}}{\lambda_{1}}-1\right]\\ \; & =n\left[\ln\left(1+\gamma_{W}\right)-\frac{\gamma_{W}}{1+\gamma_{W}}\right], \end{array}$$

The reverse divergence is

(24) $$\begin{array}{cc} D_{10} & =D\;\left(P_{1}^{n}∥P_{0}^{n}\right)=nE_{1}\;\left[\ln\frac{p_{1}\left(y\right)}{p_{0}\left(y\right)}\right]\\ \; & =n\left[\ln\frac{\lambda_{0}}{\lambda_{1}}+\frac{\lambda_{1}}{\lambda_{0}}-1\right]\\ \; & =n\left[\gamma_{W}-\ln\left(1+\gamma_{W}\right)\right]. \end{array}$$

To identify the active restriction, define $\Delta \left(\gamma_{W}\right)=D_{10}-D_{01}$. Direct subtraction and differentiation produce

(25) $$\begin{array}{cc} \Delta (\gamma_{W}) & =n\left[\gamma_{W}-2\ln\left(1+\gamma_{W}\right)+\frac{\gamma_{W}}{1+\gamma_{W}}\right],\\ \frac{d\Delta (\gamma_{W})}{d\gamma_{W}} & =\frac{n\gamma_{W}^{2}}{{\left(1+\gamma_{W}\right)}^{2}}\geq 0. \end{array}$$

Since Δ(0)=0 and its derivative is nonnegative, $D_{10}\geq D_{01}$ for every $\gamma_{W}\geq 0$. The inequality is strict for every $\gamma_{W}>0$. Equation (24) therefore determines the bidirectional KL limit.

Since the reverse divergence is dominant, the bidirectional requirement reduces to a scalar observation-SNR threshold. Let ${\bar{\gamma }}_{W}$ denote the unique nonnegative root of

(26) $$n\left[{\bar{\gamma }}_{W}-\ln\left(1+{\bar{\gamma }}_{W}\right)\right]=2\epsilon^{2}.$$

For a closed-form characterization, define the normalized KL budget as $\delta ≜2\epsilon^{2}/n$. Equation (26) then admits the exact solution

(27) $${\bar{\gamma }}_{W}=-W_{-1}\;\left(-\exp[-(1+\delta )]\right)-1,$$

where $W_{-1}\left(\cdot \right)$ is the negative branch of the Lambert *W* function. Only the negative branch produces ${\bar{\gamma }}_{W}>0$ for δ>0. For numerical evaluation, bisection recovers the same root. Its sensitivity to ϵ and *n* follows from implicit differentiation of Equation (26) as

(28) $$\begin{array}{cc} \frac{\partial {\bar{\gamma }}_{W}}{\partial \epsilon } & =\frac{4\epsilon (1+{\bar{\gamma }}_{W})}{n{\bar{\gamma }}_{W}}>0,\\ \frac{\partial {\bar{\gamma }}_{W}}{\partial n} & =-\frac{\left(1+{\bar{\gamma }}_{W}\right)\left[{\bar{\gamma }}_{W}-\ln\left(1+{\bar{\gamma }}_{W}\right)\right]}{n{\bar{\gamma }}_{W}}<0. \end{array}$$

The derivatives show that a larger tolerance raises the admissible observation SNR, whereas a longer observation interval lowers it. Hence, the bidirectional KL constraints reduce to $\gamma_{W}\leq {\bar{\gamma }}_{W}$. The sufficient condition $\gamma_{W}^{c}\leq {\bar{\gamma }}_{W}$ preserves the same KL guarantee. For a fixed Willie location, define

(29) $$\begin{array}{cc} c(q;q_{W}) & =P_{A}G_{AW}\left(q_{W}\right)-{\bar{\gamma }}_{W}\;\left[P_{J}G_{W}\left(q;q_{W}\right)+\sigma_{W}^{2}\right],\\ d(q;q_{W}) & =P_{A}MG_{A}\left(q\right)G_{W}\left(q;q_{W}\right). \end{array}$$

The sufficient condition based on Equation (19) is equivalent to

(30) $$c\left(q;q_{W}\right)+d\left(q;q_{W}\right)\beta \leq 0.$$

The coefficient $d(q;q_{W})$ is positive throughout the flight region. A location is covertness-feasible only if $c(q;q_{W})\leq 0$. Under both conditions the gain bound is

(31) $$\beta \leq \beta_{\mathrm{cov}}\left(q;q_{W}\right)≜\frac{{\bar{\gamma }}_{W}\;\left[P_{J}G_{W}\left(q;q_{W}\right)+\sigma_{W}^{2}\right]-P_{A}G_{AW}\left(q_{W}\right)}{P_{A}MG_{A}\left(q\right)G_{W}\left(q;q_{W}\right)}.$$

The numerator in Equation (31) sets the direct-link feasibility floor while omission of always-on IRS distortion preserves conservatism. Direct leakage further introduces an unknown relative phase between the direct and aggregate reflected fields. The corresponding worst-case envelope is

(32) $$\begin{array}{cc} e(q;q_{W}) & =2P_{A}\sqrt{MG_{AW}\left(q_{W}\right)G_{A}\left(q\right)G_{W}\left(q;q_{W}\right)},\\ t_{\max}\left(q;q_{W}\right) & =\frac{-e\left(q;q_{W}\right)+\sqrt{e^{2}\left(q;q_{W}\right)-4d\left(q;q_{W}\right)c\left(q;q_{W}\right)}}{2d(q;q_{W})},\\ \beta_{\mathrm{cov}}^{\mathrm{ph}}\left(q;q_{W}\right) & =t_{\max}^{2}\left(q;q_{W}\right). \end{array}$$

Equation (32) follows from the worst-case leakage $P_{A}{\left[\sqrt{G_{AW}}+\sqrt{M\beta G_{A}G_{W}}\right]}^{2}$. The envelope varies only the phase between the direct field and the root-mean-square aggregate reflected field. It does not impose element-wise coherent alignment toward Willie. The IRS elements remain phase-aligned toward Bob through Equation (11). The location-robust gain bound is

(33) $$\beta_{\mathrm{cov}}^{\mathrm{rob}}\left(q\right)=\underset{q_{W}\in U_{W}}{\inf}\;\beta_{\mathrm{cov}}^{\mathrm{ph}}\left(q;q_{W}\right),$$

provided that $c(q;q_{W})\leq 0$ for every location in $U_{W}$. A negative numerator is treated as infeasibility rather than truncation to zero. When $G_{AW}=0$, Equations (31) and (32) become identical.

The deterministic-equivalent bounds above do not extend uniformly to quasi-static Rayleigh fading with unbounded support. A guarantee over every realization would require zero reflected gain. For a blocked direct path, the instantaneous conservative Willie SNR and the covert-outage constraint are therefore defined as

(34) $$\begin{array}{cc} \gamma_{W}^{f}\left(q,\beta ;q_{W},Z\right) & =\frac{P_{A}M\beta G_{A}\left(q\right)G_{W}\left(q;q_{W}\right)Z}{P_{J}G_{W}\left(q;q_{W}\right)+\sigma_{W}^{2}},\\ p_{\mathrm{out}}\left(q,\beta \right) & ≜\underset{q_{W}\in U_{W}}{\sup}\mathrm{Pr}\;\left\{n\;\left[\gamma_{W}^{f}-\ln\left(1+\gamma_{W}^{f}\right)\right]>2\epsilon^{2}\right\}\leq p_{\mathrm{out}}^{\max}. \end{array}$$

The probability in Equation (34) is taken across independent coherence blocks. The notation $p_{\mathrm{out}}^{\max}$ separates the admissible violation probability from the normalized KL budget δ. With independent Rayleigh cascaded leakage and a deterministic jammer denominator, define the normalized leakage factor

(35) $$Z=\frac{1}{M}{\left|\sum_{m=1}^{M}g_{W,m}^{*}e^{j\phi_{m}^{★}}g_{A,m}\right|}^{2}.$$

Circular symmetry of $g_{W,m}$ makes the distribution of *Z* independent of the Bob-directed phase vector. The closed-form tail applies to the calibrated equal-gain response $r_{m}\left(\phi \right)=1$. Its finite-array tail probability is

(36) $$\mathrm{Pr}\left\{Z>z\right\}=\frac{2{\left(Mz\right)}^{M/2}K_{M}\;\left(2\sqrt{Mz}\right)}{\Gamma (M)},$$

where $K_{M}\left(\cdot \right)$ is the modified Bessel function of the second kind. Let $\kappa_{M}$ satisfy $\mathrm{Pr}\left\{Z>\kappa_{M}\right\}=p_{\mathrm{out}}^{\max}$. When the direct path is blocked and the covertness bound is active, the chance-constrained gain is

(37) $$\beta_{\mathrm{cov}}^{\mathrm{cc}}\left(q\right)=\frac{\beta_{\mathrm{cov}}^{\mathrm{rob}}\left(q\right)}{\kappa_{M}}.$$

Equation (37) provides an exact finite-*M* safety factor for the stated fading model. A random jammer channel changes the denominator distribution and falls outside that closed form. Section 5 therefore evaluates jammer fading through block-level Monte Carlo trials at the reference UAV location. Nonzero direct leakage requires a numerical quantile of Equation (34) unless the deterministic relative phase bound is adopted. For a blocked direct path, Equation (37) replaces $\beta_{\mathrm{cov}}^{\mathrm{rob}}$ within the chance-constrained placement search. The deterministic formulation retains $\beta_{\mathrm{cov}}^{\mathrm{rob}}$.

## 4. Joint UAV Placement and Active-IRS Power Gain Optimization

In addition to covertness, the active IRS obeys an output budget with average power

(38) $$\begin{array}{cc} P_{\mathrm{IRS}}\left(q,\beta \right) & =E\;\left[{∥\Theta \left(\sqrt{P_{A}}h_{A}s+{\tilde{n}}_{U}\right)∥}_{2}^{2}\right]\\ \; & =M\beta \left[P_{A}G_{A}\left(q\right)+\nu_{U}\right]. \end{array}$$

The hardware requirement $P_{\mathrm{IRS}}\leq P_{\max}$ therefore imposes

(39) $$\beta \leq \beta_{\mathrm{pow}}\left(q\right)≜\frac{P_{\max}}{M\left[P_{A}G_{A}\left(q\right)+\nu_{U}\right]}.$$

Define $F≜\{q_{h}\in Q:c\left(q;q_{W}\right)\leq 0\;\mathrm{for}\;\mathrm{every}\;q_{W}\in U_{W}\}$. This set contains every location that admits a nonnegative covertness-feasible gain in the continuous Willie uncertainty region. The hardware and covertness limits jointly yield

(40) $$\begin{array}{cc} \underset{q_{h}\in F,\;\beta \geq 0}{\mathrm{maximize}}\;\; & \gamma_{B}\left(q,\beta \right)\\ \mathrm{subject}\;\mathrm{to}\;\; & \beta \leq \beta_{\mathrm{cov}}^{\mathrm{rob}}\left(q\right),\\ \; & P_{\mathrm{IRS}}\left(q,\beta \right)\leq P_{\max}. \end{array}$$

For calibrated equal-gain elements, Equation (11) solves the phase subproblem at every candidate location. The closed-form update leaves only the UAV position and common power gain.

At a fixed UAV location with $A_{\mathrm{hw}}>0$, define $A\left(q\right)=P_{A}A_{\mathrm{hw}}G_{A}\left(q\right)G_{B}\left(q\right)$ and $B\left(q\right)=M\nu_{U}G_{B}\left(q\right)$. The nominal phase solution corresponds to $A_{\mathrm{hw}}=M^{2}$ while $C\left(q\right)=\rho_{B}P_{J}G_{B}\left(q\right)+\sigma_{B}^{2}$ collects the remaining denominator terms. Equation (12) then reduces to $\gamma_{B}=A\beta /\left(B\beta +C\right)$ with derivative

(41) $$\frac{\partial \gamma_{B}\left(q,\beta \right)}{\partial \beta }=\frac{A(q)C(q)}{{\left[B(q)\beta +C(q)\right]}^{2}}>0.$$

Since the derivative is positive throughout the feasible region, Bob’s SINR increases strictly with β. Hence, the pointwise optimal gain is

(42) $$\beta^{★}\left(q\right)=\min\left\{\beta_{\mathrm{cov}}^{\mathrm{rob}}\left(q\right),\beta_{\mathrm{pow}}\left(q\right)\right\}.$$

The aggregate output budget sets the admissible gain for the considered amplifier model. A device-specific element gain cap enters the minimum in Equation (42) without changing the placement procedure. Equation (42) further identifies whether covertness or output power limits each feasible location. The corresponding regions are

(43) $$\begin{array}{cc} Q_{\mathrm{cov}} & ≜\left\{q_{h}\in F:\beta_{\mathrm{cov}}^{\mathrm{rob}}\left(q\right)\leq \beta_{\mathrm{pow}}\left(q\right)\right\},\\ Q_{\mathrm{pow}} & ≜\left\{q_{h}\in F:\beta_{\mathrm{pow}}\left(q\right)<\beta_{\mathrm{cov}}^{\mathrm{rob}}\left(q\right)\right\}. \end{array}$$

Their switching boundary is

(44) $$C=\left\{q_{h}\in F:\beta_{\mathrm{cov}}^{\mathrm{rob}}\left(q\right)=\beta_{\mathrm{pow}}\left(q\right)\right\}.$$

Substitution of Equation (42) into Equation (12) produces

(45) $${\tilde{\gamma }}_{B}\left(q\right)=\left\{\begin{array}{cc} \frac{A\left(q\right)\beta_{\mathrm{cov}}^{\mathrm{rob}}\left(q\right)}{B\left(q\right)\beta_{\mathrm{cov}}^{\mathrm{rob}}\left(q\right)+C\left(q\right)},\; & q_{h}\in Q_{\mathrm{cov}},\\ \frac{A\left(q\right)\beta_{\mathrm{pow}}\left(q\right)}{B\left(q\right)\beta_{\mathrm{pow}}\left(q\right)+C\left(q\right)},\; & q_{h}\in Q_{\mathrm{pow}}. \end{array}\right.$$

For the covertness-limited branch, direct leakage remains coupled to the reflected Willie link. With the power-limited branch, the IRS output budget determines the gain. Both branches retain waveform-cancellation error through C(q) and jammer-to-IRS coupling through $\nu_{U}$.

Mixed LoS and NLoS gains make the reduced objective nonconvex in the horizontal position. The minimum in Equation (42) further introduces a location-dependent switching boundary. Nevertheless, the closed-form phase and gain solutions leave only two horizontal variables. For a singleton $U_{W}$, an exhaustive finite-grid search avoids dependence on initialization and returns the exact maximizer over $Q_{\Delta }\cap F$. Under continuous uncertainty conditions, the same search uses certified lower gain bounds at tolerance $\tau_{U}$.

Accordingly, define the coordinate sets $X={\left\{x_{\min}+i\Delta_{x}\right\}}_{i=0}^{N_{x}-1}$ and $Y={\left\{y_{\min}+j\Delta_{y}\right\}}_{j=0}^{N_{y}-1}$. Their Cartesian product is $Q_{\Delta }=X\times Y$. The finite-grid placement problem is

(46) $$q_{h}^{\Delta }\in \underset{q_{h}\in Q_{\Delta }\cap F}{\mathrm{arg max}}\;{\tilde{\gamma }}_{B}\left(q\right).$$

The numerical procedure first computes ${\bar{\gamma }}_{W}$ from Equation (26). For each UAV grid point, a two-dimensional interval branch-and-bound search evaluates Equation (33) over the continuous uncertainty set. Interval extensions enclose *c* and $\beta_{\mathrm{cov}}^{\mathrm{ph}}$ on every active subregion. A nonpositive upper enclosure of *c* certifies the condition c≤0, whereas a positive lower enclosure proves infeasibility. Unresolved subregions are bisected along their widest coordinates. The smallest gain lower bound and the smallest sampled gain form a global enclosure of $\beta_{\mathrm{cov}}^{\mathrm{rob}}$. The inner search terminates once its enclosure gap is no larger than $\tau_{U}$ and every active subregion has passed the feasibility test. Its certified lower bound then enters Equation (42) before phase and SINR evaluation. The outer grid search retains the best feasible tuple. Algorithm 1 summarizes the complete procedure.
**Algorithm 1** Robust joint UAV placement and active-IRS design**Require:** 
X, Y, *H*, $U_{W}$, $\tau_{U}$, and system parameters**Ensure:** 
A feasible tuple $\left(q^{\Delta },\beta^{\Delta },\phi^{\Delta },\gamma_{B}^{\Delta }\right)$ or an infeasibility declaration  1:**Phase 1: Covertness threshold**  2:Solve Equation (26) for ${\bar{\gamma }}_{W}$  3:**Phase 2: Robust grid evaluation**  4:Set $\gamma_{B}^{\Delta }\leftarrow -\infty$ and $f^{\Delta }\leftarrow 0$  5:**for** each (x,y)∈X×Y **do**  6:   Set $q\leftarrow {\left[x,y,H\right]}^{T}$ and evaluate $G_{A}$ and $G_{B}$  7:   Initialize the interval subregion list with $U_{W}$  8:   Enclose *c* and $\beta_{\mathrm{cov}}^{\mathrm{ph}}$ on every active subregion  9:   Bisect unresolved subregions until infeasibility is proved or the certified gain gap does not exceed $\tau_{U}$10:   **if** c≤0 is not certified throughout $U_{W}$ **then**11:      Reject q and continue the grid search12:   **end if**13:   Set $\beta_{\mathrm{cov}}^{\mathrm{rob}}$ to the certified global lower gain bound14:   Evaluate $\beta_{\mathrm{pow}}$ through Equation (39)15:   Set $\beta \leftarrow \min\{\beta_{\mathrm{cov}}^{\mathrm{rob}},\beta_{\mathrm{pow}}\}$ through Equation (42)16:   Evaluate $\phi^{★}$ through Equation (11) and $\gamma_{B}$ through Equation (12)17:   **if** $\gamma_{B}>\gamma_{B}^{\Delta }$ **then**18:      Set $\left(q^{\Delta },\beta^{\Delta },\phi^{\Delta },\gamma_{B}^{\Delta }\right)\leftarrow \left(q,\beta ,\phi^{★},\gamma_{B}\right)$19:      Set $f^{\Delta }\leftarrow 1$20:   **end if**21:**end for**22:**Phase 3: Solution return**23:**if **$f^{\Delta }=0$** then**24:   **return** Infeasibility25:**end if**26:**return **$q^{\Delta }$, $\beta^{\Delta }$, $\phi^{\Delta }$, and $\gamma_{B}^{\Delta }$

Let $N_{U}\left(\tau_{U}\right)$ denote the number of subregions visited by the uncertainty solver at one grid point. Constant-cost interval evaluation leads to $C_{U}\left(\tau_{U}\right)=O\left[N_{U}\left(\tau_{U}\right)\right]$. A maximum binary subdivision depth $L_{U}$ has the worst-case bound $N_{U}=O\left(2^{L_{U}}\right)$. With $K_{r}$ scalar-root iterations, the total complexity is $O\{K_{r}+N_{x}N_{y}\left[C_{U}\left(\tau_{U}\right)+M\right]\}$. A singleton uncertainty set reduces $C_{U}$ to O(1) and the total complexity to $O(K_{r}+N_{x}N_{y}M)$. Best-point storage requires constant search memory apart from the branch-and-bound state and *M* phase coefficients. A measured amplitude response adds the numerical phase-solver cost at each grid point. If no certified point exists, the procedure returns an infeasibility declaration.

Finally, exact execution at a grid waypoint satisfies the pointwise constraints. A cell-robust extension covers horizontal navigation error at altitude *H*. For $q={\left[q_{h}^{T},H\right]}^{T}$, define the navigation cell and its admissible index set as

(47) $$\begin{array}{c} C_{g}=\left\{q_{h}={\left[x,y\right]}^{T}\in Q:|x-x_{g}|\leq \frac{\Delta_{x}}{2},\;\left|y-y_{g}\right|\leq \frac{\Delta_{y}}{2}\right\},\\ I_{\mathrm{cell}}=\left\{g:C_{g}\subseteq Q,\;c\left(q;q_{W}\right)<0\;\mathrm{for}\;\mathrm{every}\;q_{h}\in C_{g}\;\mathrm{and}\;q_{W}\in U_{W}\right\}. \end{array}$$

For every $g\in I_{\mathrm{cell}}$ the assigned gain is

(48) $$\beta_{g}^{\mathrm{cell}}=\left(1-\zeta_{g}\right)\underset{q_{h}\in C_{g}}{\inf}\;\min\left\{\beta_{\mathrm{cov}}^{\mathrm{rob}}\left(q\right),\beta_{\mathrm{pow}}\left(q\right)\right\},\;0<\zeta_{g}<1.$$

The interval procedure evaluates $C_{g}\times U_{W}$ and rejects every index outside $I_{\mathrm{cell}}$. The factor $\zeta_{g}$ reserves a positive numerical margin below each limiting gain. Equation (48) therefore preserves strict covertness and the output-power constraint throughout $C_{g}$. The pointwise limit $\zeta_{g}↓0$ with a singleton cell recovers Equation (42).

## 5. Simulation Results

### 5.1. Simulation Settings

Table 1 summarizes the baseline parameters. All power values are converted from dBm to watts before numerical evaluation. With the deterministic gains in Equation (5), Equation (26) yields ${\bar{\gamma }}_{W}=3.166\times 10^{-3}$. The conservative baseline assumes a blocked Alice-to-Willie path and perfect suppression at Bob and the IRS. A singleton uncertainty set specifies $U_{W}=\left\{q_{W}\right\}$. With this specialization, Equation (19) omits only the masking contribution of IRS circuit noise. One robustness test introduces quasi-static leakage peaks through the finite-array chance constraint. Separate sweeps activate direct leakage and residual interference. Every reported point satisfies the applicable output-power and covertness restrictions.

### 5.2. UAV Placement and Architecture Comparison

**Figure 2:**
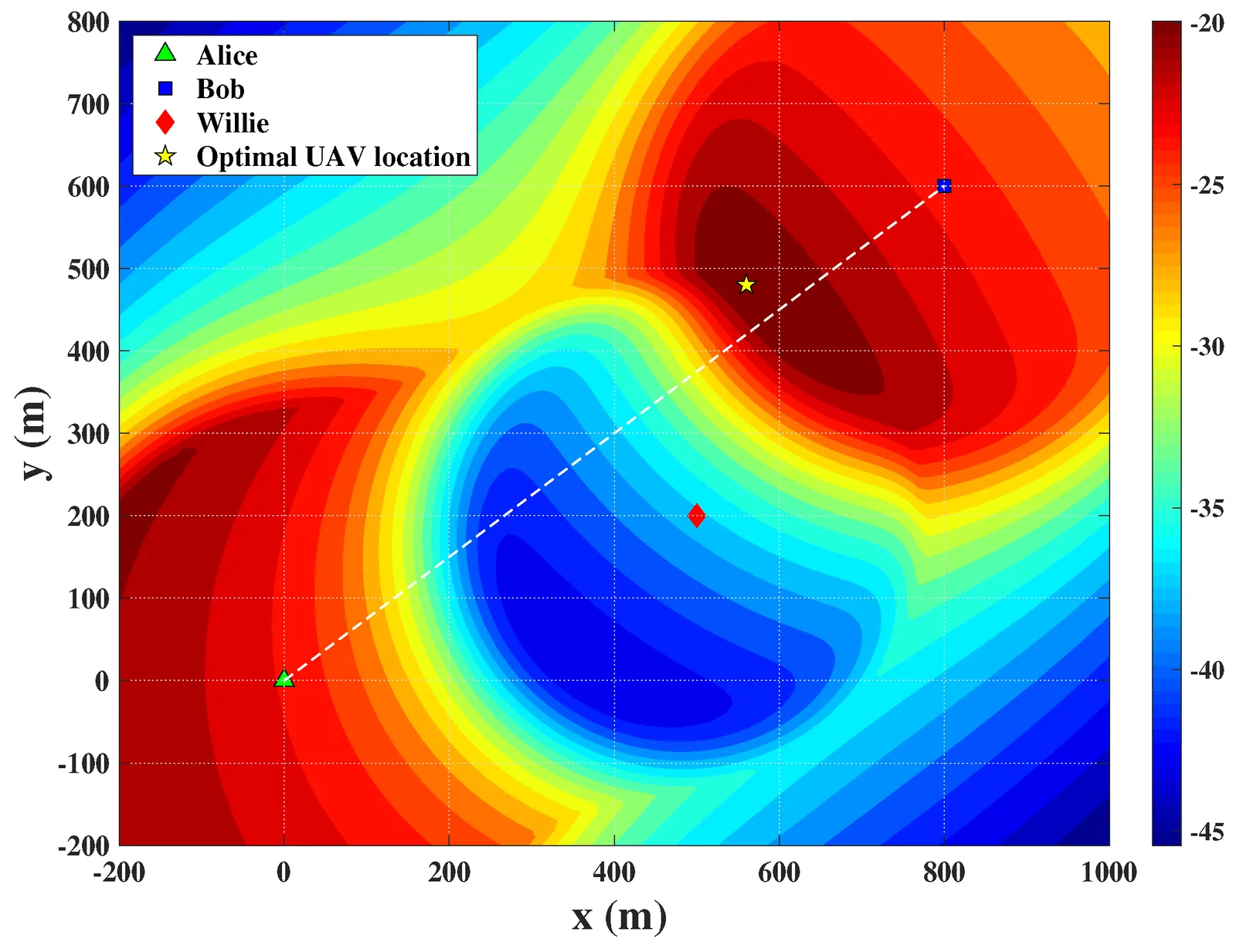
Bob SNR landscape over the UAV horizontal search region.

Figure 2 maps Bob’s SNR over the baseline flight region and identifies $q^{★}=\left(560,480,100\right)$ m with an SNR of −18.69 dB. Suppression of the admissible gain near Willie creates a pronounced valley, whereas locations near Alice or Bob benefit from a stronger cascaded hop. A large $G_{B}$ favors the region near Bob by reducing the relative effect of forwarded circuit noise in Equation (12). Nevertheless, the optimum remains separated from Bob due to the coupled Alice-to-UAV loss and Willie leakage. Its displacement from the direct Alice-to-Bob line further shows that shortest-path geometry does not determine the covert optimum. Joint optimization of gain and placement is therefore essential across the flight region.

**Figure 3:**
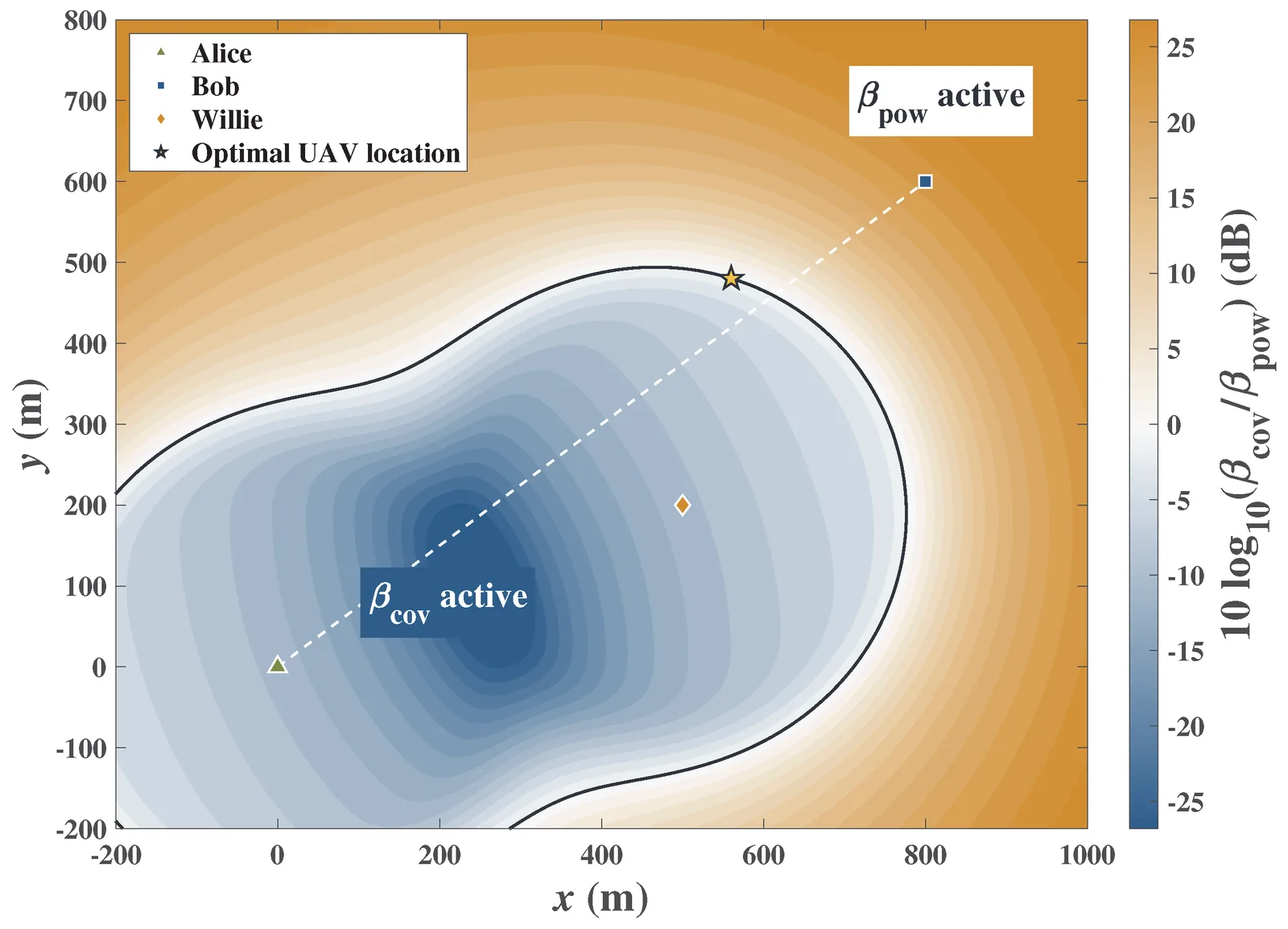
Spatial distribution of the active power-gain constraint over the UAV placement region.

To identify the mechanism behind the SNR landscape, Figure 3 compares the covertness and output-power gain bounds through $10\log_{10}\left(\beta_{\mathrm{cov}}/\beta_{\mathrm{pow}}\right)$. Negative values mark covertness-limited locations, whereas positive values mark power-limited locations. The zero contour is the switching boundary in Equation (44). Covertness and output power govern 43.5% and 56.5% of the sampled grid.

The covertness-limited region surrounds Willie and extends toward Alice since a stronger $G_{A}$ increases reflected leakage. By contrast, the output budget governs the outer region near Bob and the upper flight boundary. At the optimum, $10\log_{10}\left(\beta_{\mathrm{cov}}/\beta_{\mathrm{pow}}\right)=-0.046$ dB. The corresponding $\beta_{\mathrm{cov}}$ is only 1.06% below $\beta_{\mathrm{pow}}$. A move toward Willie tightens covertness, whereas a move farther into the power-limited region weakens the cascaded channel. Consequently, the switching neighborhood balances detection exposure against available IRS output power. The map further explains why one fixed gain is unsuitable across the complete flight region.

**Figure 4:**
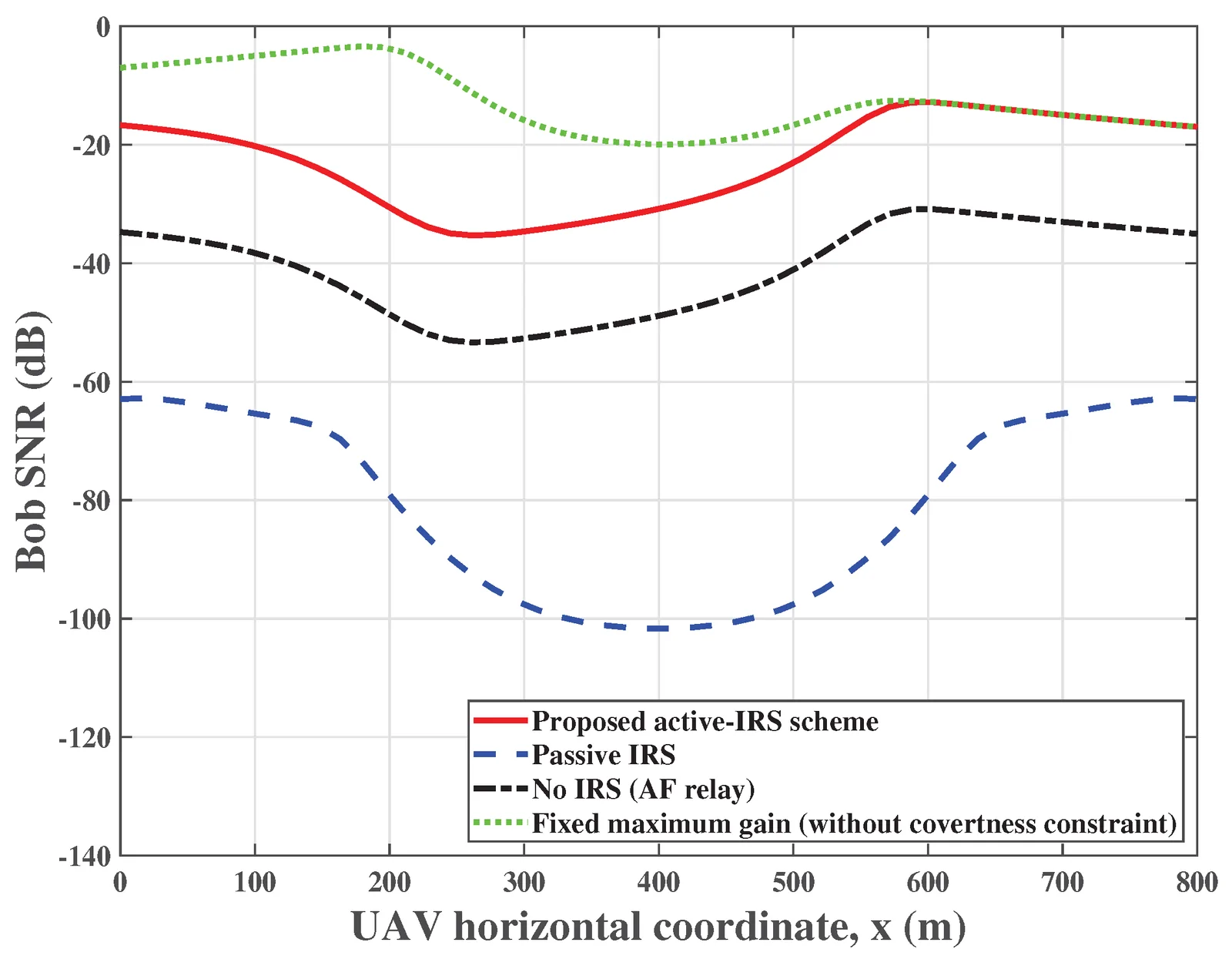
Bob SNR along the Alice-to-Bob UAV trajectory with four relaying architectures.

Figure 4 compares four architectures along a straight Alice-to-Bob UAV trajectory with x∈[0,800] m and y=0.75x. At each trajectory point, the proposed active IRS applies Equation (42) with M=64. The passive IRS restricts β≤1 and excludes circuit noise, whereas the amplify-and-forward relay uses M=1. The fixed-gain reference applies $\beta_{\mathrm{pow}}$ without a covertness limit and forms an upper reference wherever its SNR exceeds the feasible active-IRS curve.

When covertness controls amplification, the proposed curve separates from the fixed-gain reference. Both active-IRS curves coincide near Bob after the output-power bound becomes active. Although coherent phase alignment benefits the passive IRS, its unit gain does not overcome the cascaded path loss. The single-element relay avoids passive attenuation and lacks the $M^{2}$ coherent array factor. Simultaneously weak Alice-to-UAV and UAV-to-Bob links produce the deep central minima. Active amplification instead preserves a substantial SNR advantage throughout the trajectory.

### 5.3. Robustness to Direct Leakage and Small-Scale Fading

**Figure 5:**
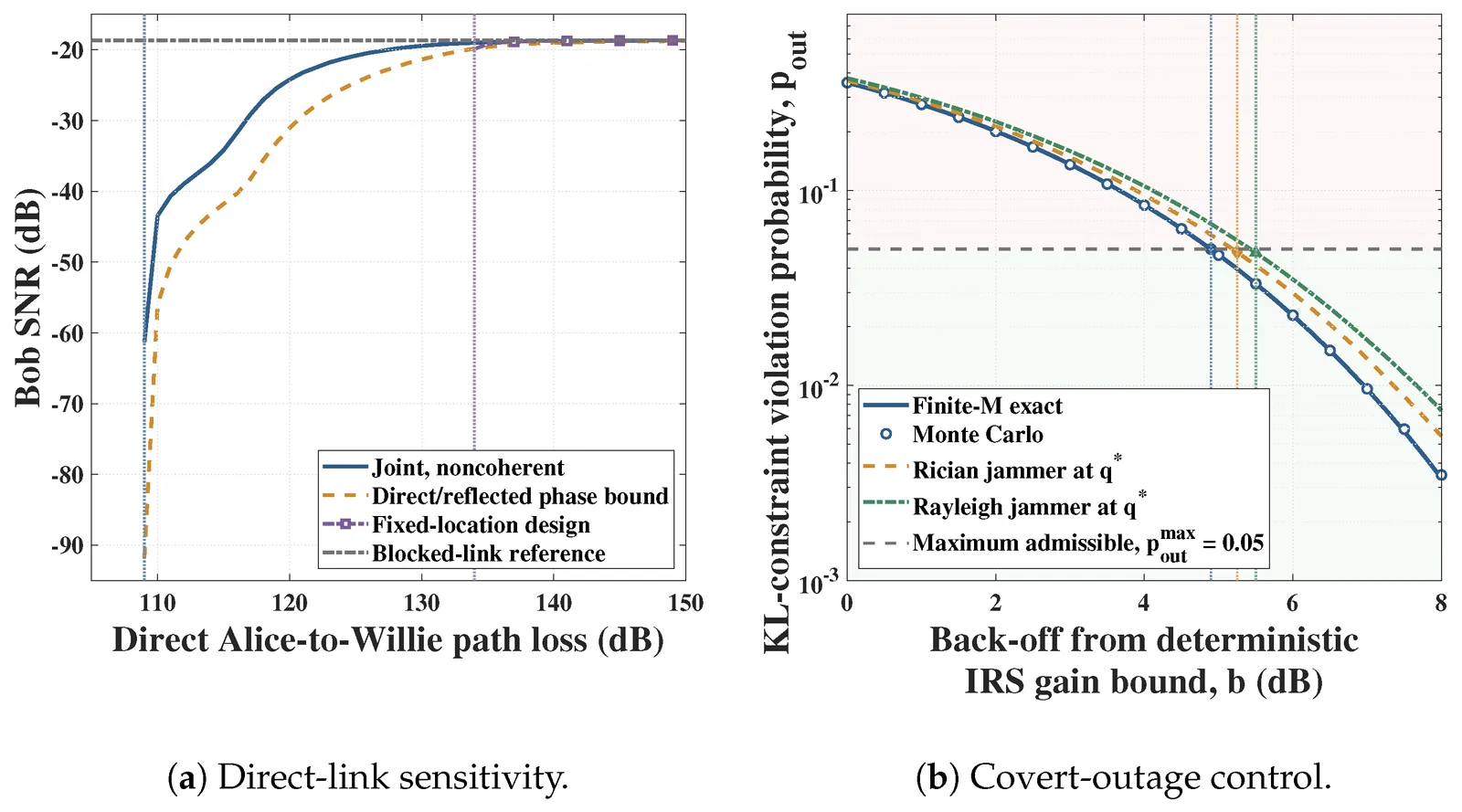
Propagation robustness under direct leakage and quasi-static small-scale fading conditions.

Figure 5a evaluates robustness after activation of the direct Alice-to-Willie path. At each terrestrial path loss, the joint designs reoptimize UAV placement and active gain. The noncoherent and relative-phase curves apply Equations (16) and (32). The fixed-location design retains the baseline UAV position and updates only its gain. Direct-link leakage excludes every candidate below 109 dB, whereas the fixed position first becomes feasible near 134 dB. Joint placement therefore extends the feasible covert range by 25 dB. The relative-phase bound remains below its noncoherent counterpart and approaches the blocked-link reference as $L_{AW}$ increases.

Figure 5b tests the finite-array chance constraint in Equation (34). The horizontal variable $b=-10\log_{10}\left(\beta /\beta_{\mathrm{cov}}\right)$ measures back-off from the deterministic large-scale bound. Points above $p_{\mathrm{out}}^{\max}=0.05$ are rejected trials rather than outputs of the robust design. The exact finite-*M* tail in Equation (36) agrees with $3\times 10^{5}$ independent Monte Carlo blocks. For M=16, the exact tail requires b=4.897 dB. Rician and Rayleigh jammer fading shift the first safe 0.25 dB grid points to 5.25 dB and 5.50 dB. Each threshold uses a pointwise one-sided 95% Wilson upper bound with independent jammer-link and cascaded-leakage samples. The finite-array back-off moves the reoptimized UAV from (560,480,100) m to (550,510,100) m with an SNR cost of only 0.271 dB. Accordingly, every adopted setting lies on the admissible side of the horizontal threshold.

### 5.4. Residual Interference and Circuit-Noise Sensitivity

**Figure 6:**
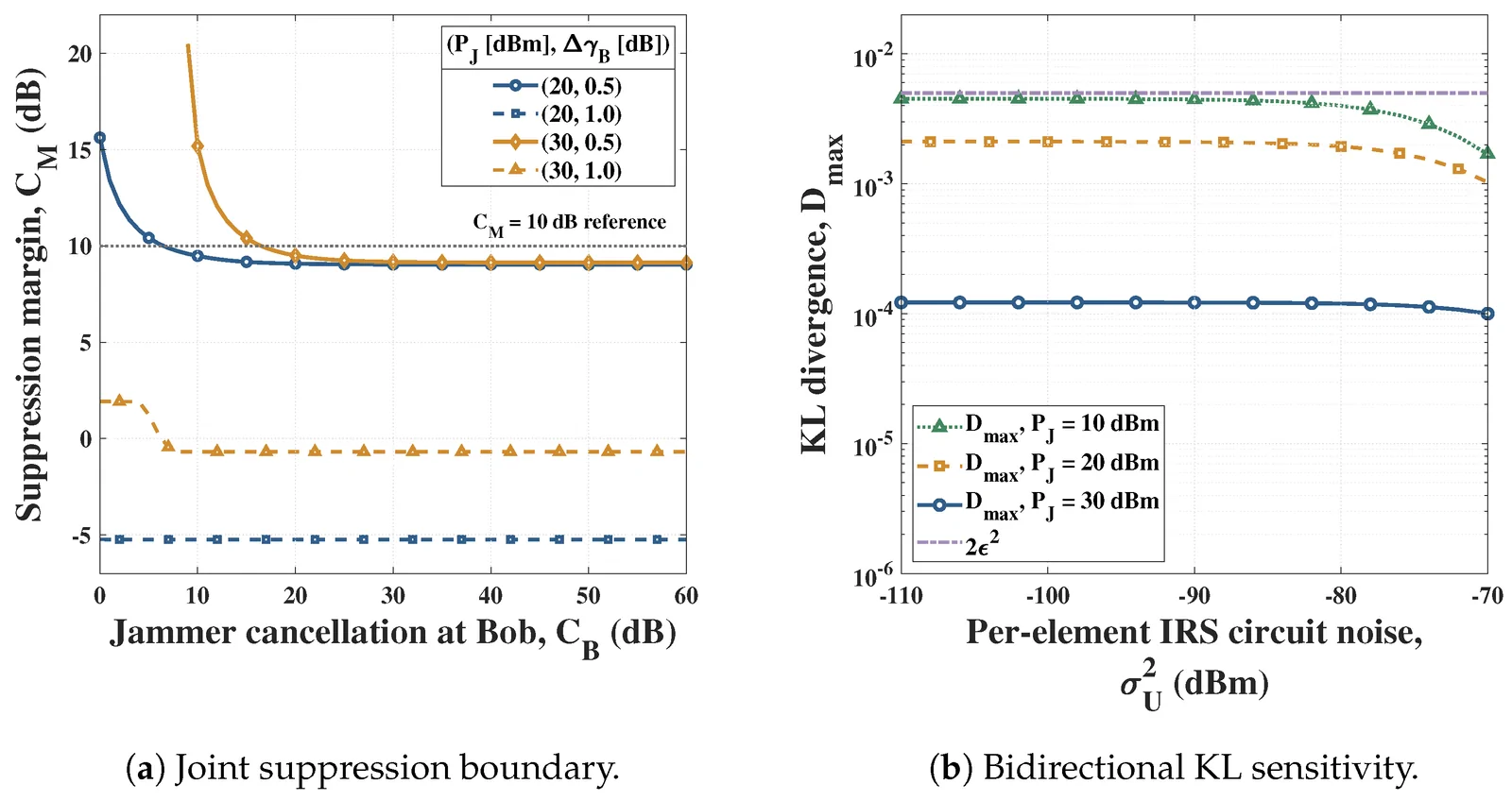
Hardware and detector sensitivity with residual friendly-jammer coupling and always-on IRS circuit noise.

Figure 6a maps the joint suppression required at Bob and the active IRS. Here, $C_{M}=10\log_{10}\left[\sigma_{U}^{2}/\left(\eta_{\mathrm{SI}}P_{J}\right)\right]$ measures residual jammer coupling relative to the circuit-noise floor. Each boundary follows joint reoptimization of UAV placement and active gain. A point above a boundary limits the Bob-SINR degradation to the corresponding $\Delta \gamma_{B}$. At $P_{J}=30$ dBm and $C_{B}=10$ dB the 0.5 dB target requires $C_{M}=15.18$ dB. An increase in $C_{B}$ to 18 dB reduces the requirement to 9.73 dB. For $P_{J}=20$ dBm the analogous boundary reaches 9.72 dB at $C_{B}=8$ dB. Absent low-$C_{B}$ segments indicate that residual jamming at Bob already violates the target under ideal jammer-to-IRS isolation conditions. The curves therefore specify a joint hardware condition without presuming perfect rejection at a single-antenna receiver.

By contrast, Figure 6b isolates always-on IRS noise through the exact bidirectional divergence computed from the variances in Equation (17). The UAV remains at $q^{★}$ with $L_{AW}=140$ dB. Equation (32) imposes the worst relative phase, whereas $\eta_{\mathrm{SI}}=0$ isolates $\sigma_{U}^{2}$. A fixed reference gain applies the relative-phase bound at $P_{J}=10$ dBm with a 0.5 dB back-off. That gain respects the 1 W output budget throughout the sweep. Every $D_{\max}=\max\left\{D_{10},D_{01}\right\}$ curve remains below $2\epsilon^{2}$. The maximum divergence is $4.512\times 10^{-3}$ at $\sigma_{U}^{2}=-110$ dBm and remains 9.75% below the limit. Stronger jamming reduces divergence through a larger common variance under both hypotheses. Additional circuit noise raises the same variance and consumes the IRS output budget. The maximum output power is 0.124 W across the displayed domain. Figure 6b therefore verifies the conservatism of Equation (19) with a feasible fixed gain.

### 5.5. Effect of UAV Altitude

**Figure 7:**
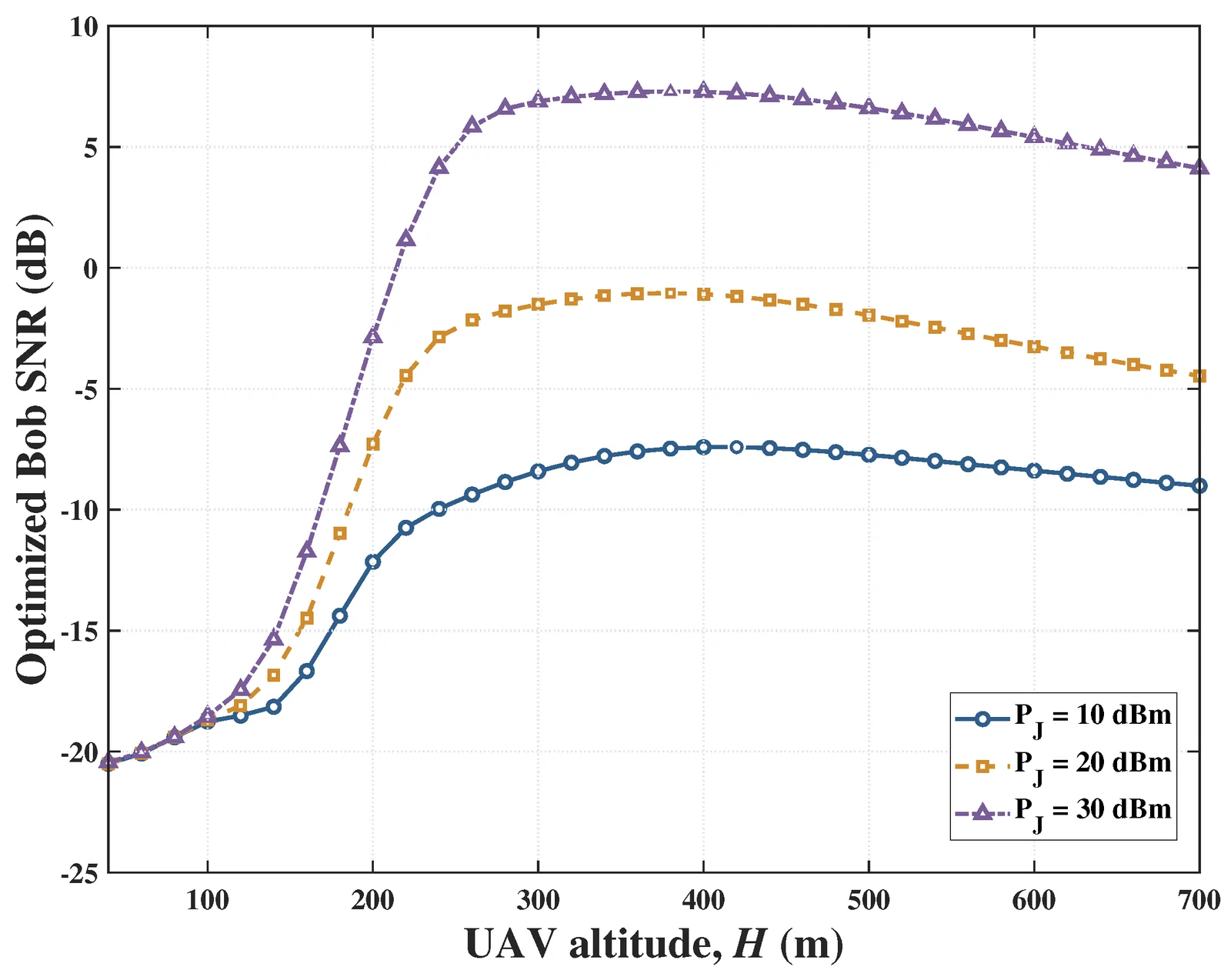
Optimized Bob SNR versus UAV altitude for three jamming powers.

Figure 7 evaluates vertical sensitivity over H∈[40,700] m. At each altitude, horizontal placement and active gain are reoptimized over the baseline grid. The curves use $P_{J}\in \left\{10,20,30\right\}$ dBm with all remaining parameters from Table 1. The comparison therefore isolates vertical geometry and friendly jamming under joint horizontal adaptation conditions.

At H=40 m, all curves lie between −20.50 dB and −20.44 dB. The UAV-to-Willie link is too weak for $P_{J}G_{W}$ to rival $\sigma_{W}^{2}$ in that regime. Consequently, a 20 dB jamming-power variation has little influence. A higher altitude increases the LoS probabilities and strengthens the masking contribution at Willie. The cascaded links improve until three-dimensional distance becomes the dominant loss mechanism.

The 10 dBm curve peaks at −7.41 dB and H=420 m. Both curves for stronger jamming peak at H=380 m with values of −1.04 dB and 7.29 dB. An increase in $P_{J}$ from 10 dBm to 20 dBm raises the peak by 6.37 dB. A further 10 dB increase raises it by 8.34 dB. At H=700 m the 10 dBm curve lies 1.60 dB below its peak. The corresponding losses are 3.42 dB and 3.17 dB for 20 dBm and 30 dBm. Stronger jamming shifts the preferred altitude from 420 m to 380 m as covertness relief becomes effective at lower elevation. The shared 380 m optimum indicates that additional jamming changes SNR more strongly than altitude after the LoS probabilities saturate. Interior maxima therefore establish the value of joint vertical and horizontal placement.

### 5.6. Effect of the Covertness Tolerance

**Figure 8:**
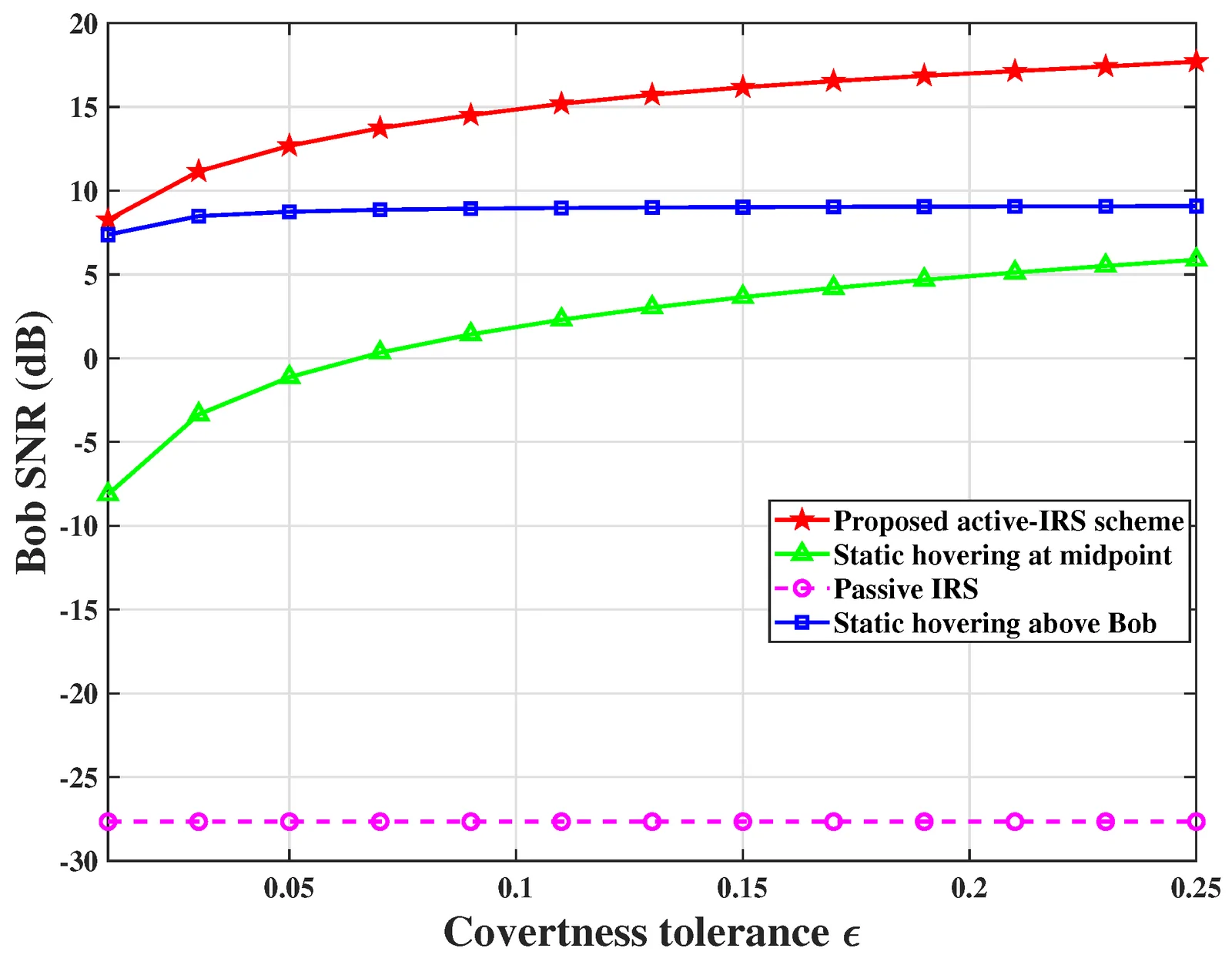
Bob SNR versus covertness tolerance for active and passive IRS schemes.

Figure 8 evaluates covertness tolerance under $q_{A}=\left(0,0,0\right)$ m and $q_{B}=\left(500,0,0\right)$ m. Willie is located at (480,20,0) m. The test sets $P_{A}=P_{\max}=45$ dBm and M=64 over a 20 m placement grid. Its horizontal region is x∈[0,600] m and y∈[−100,100] m. The proposed scheme jointly optimizes placement and active gain. Both fixed-location schemes optimize only their gains, whereas the passive benchmark optimizes placement under β≤1.

The proposed scheme attains the highest SNR throughout the tested tolerance range. An increase in ϵ raises ${\bar{\gamma }}_{W}$ through Equation (26) and relaxes Equation (31). Active designs consequently admit stronger reflection. Joint placement converts the relaxed limit into continued SNR growth by adapting the balance between cascaded loss and leakage. In comparison, the UAV above Bob reaches its hardware-limited region at a smaller tolerance and approaches saturation. The midpoint curve grows more gradually with fixed geometry. The passive benchmark remains insensitive to ϵ under the binding condition β=1. The widening gap from the proposed curve confirms the benefit of joint placement under relaxed covertness conditions.

### 5.7. Effects of IRS Size and Jamming Power

**Figure 9:**
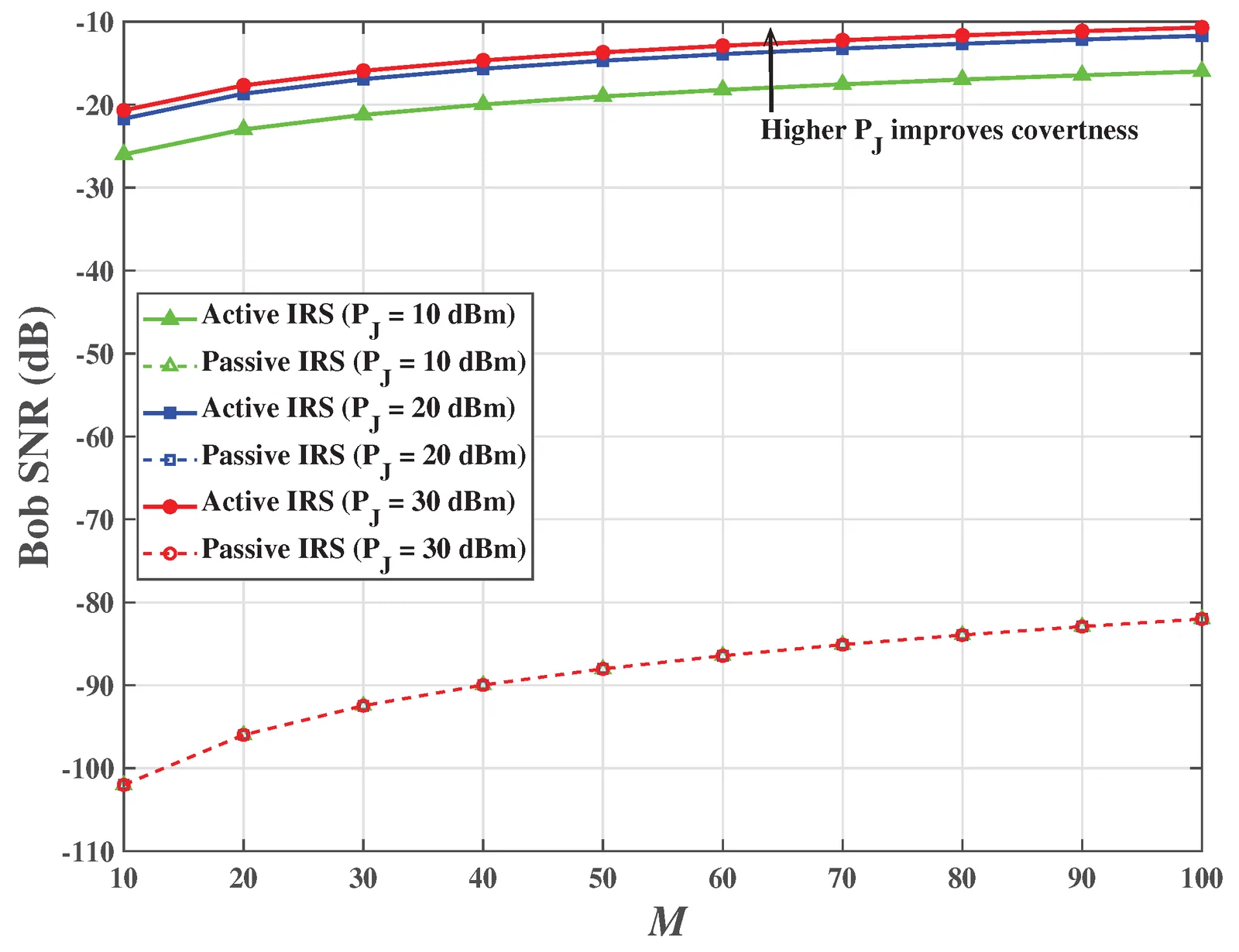
Bob SNR versus IRS element count with three jamming powers.

Figure 9 evaluates array scaling at the fixed UAV location (600,400,100) m with $P_{A}=P_{\max}=30$ dBm. The test uses ϵ=0.05 and M∈[10,100] at three jamming powers. Both $\beta_{\mathrm{cov}}$ and $\beta_{\mathrm{pow}}$ scale inversely with *M* at that location. Consequently, the coherent term $M^{2}\beta$ retains a net array gain while the distortion factor Mβ remains bounded. Bob’s SNR increases with *M* for every curve and the active designs gain over 9 dB across the tested range. A larger $P_{J}$ raises the covertness-limited gain until the output budget controls amplification. The proximity of the 20 dBm and 30 dBm active curves identifies that transition. Passive curves overlap under β=1 for every jamming power. Their 20 dB array gain remains insufficient for overcoming severe cascaded attenuation.

### 5.8. Effects of Willie Distance and Power Budget

**Figure 10:**
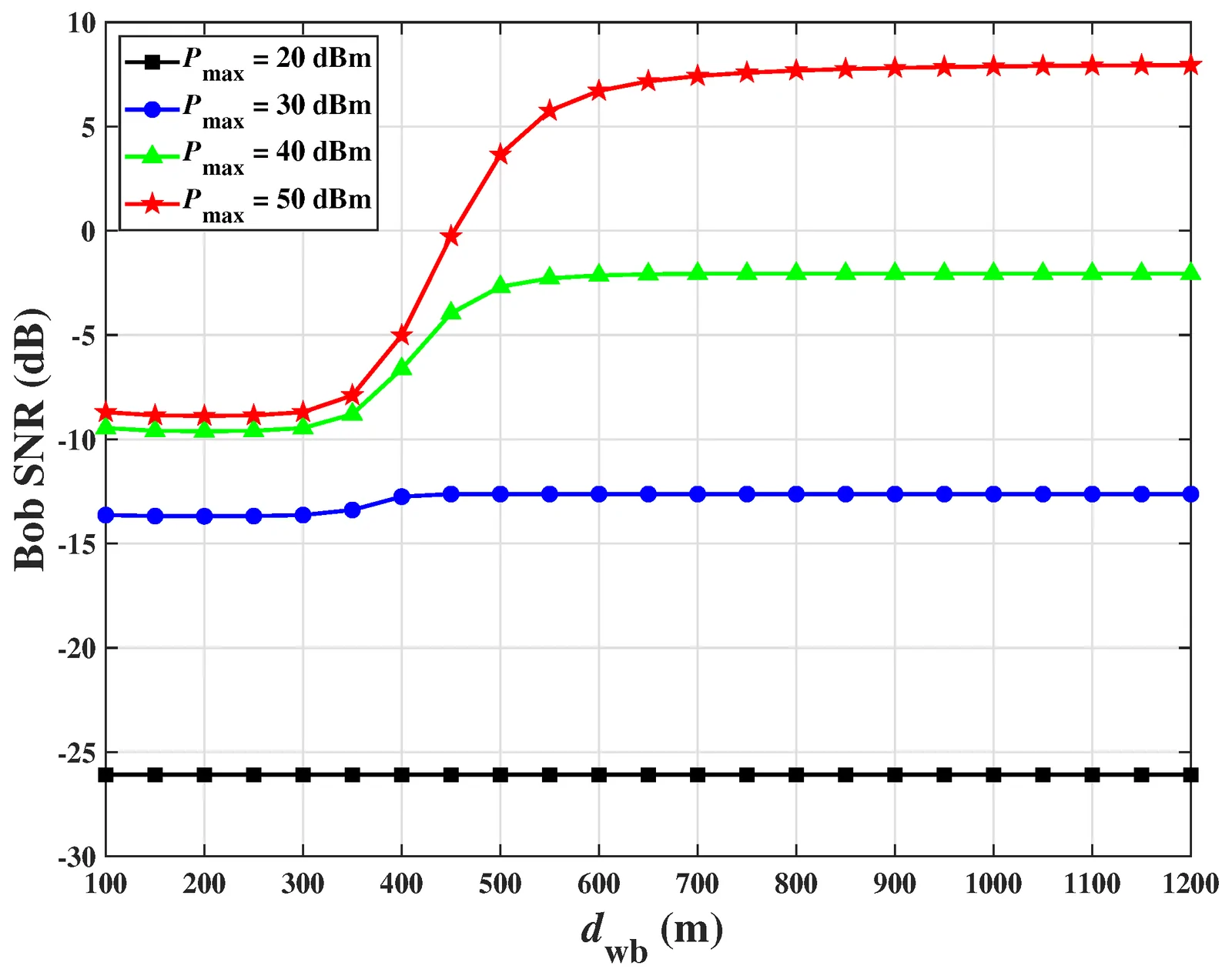
Bob SNR versus Willie-to-Bob distance under common source and IRS power budgets.

Figure 10 evaluates Willie-to-Bob distance with the UAV fixed at (600,400,100) m and M=64. Willie follows $q_{W}=q_{B}-{\left[d_{WB},0,0\right]}^{T}$ for $d_{WB}\in \left[50,1200\right]$ m. Each curve sets $P_{A}=P_{\max}\in \left\{20,30,40,50\right\}$ dBm with $P_{J}=20$ dBm and ϵ=0.05.

The 20 dBm curve is power-limited throughout the sweep, whereas the 30 dBm curve exhibits only weak geometric variation. Higher budgets reveal a transition from covertness-limited to power-limited operation. Although $d_{WB}$ increases monotonically, the UAV-to-Willie distance first decreases and reaches its minimum at $d_{WB}=200$ m. That geometry explains the initial SNR decline under the 40 dBm and 50 dBm budgets. Beyond 200 m Willie recedes from the UAV and permits a larger $\beta_{\mathrm{cov}}$. Each curve reaches a plateau once $\beta_{\mathrm{pow}}$ becomes active. A larger common source and IRS budget raises the plateau without changing the underlying constraint transition.

## 6. Conclusions

We have developed a UAV-assisted active-IRS architecture that supports covert communication over a blocked ground link. The proposed model has retained direct Alice-to-Willie leakage and bounded uncertainty in Willie’s large-scale location information. Protected waveform cancellation has replaced ideal spatial isolation at Bob, while an always-on IRS model has incorporated circuit noise and residual jammer coupling. Furthermore, bidirectional KL analysis has identified the reverse divergence as the tighter restriction and has produced a conservative gain bound under relative phase uncertainty conditions. Closed-form phase control has eliminated the calibrated equal-gain phase variables, while gain monotonicity has reduced the remaining design to a finite placement search. A finite-array chance constraint has established a model-specific back-off with a prescribed covert-outage probability. Numerical results have demonstrated a SINR advantage over passive reflection and single-element relaying across the evaluated settings. They have further quantified the direct-link feasibility boundary and the joint suppression requirements at Bob and the IRS. Overall, the findings have clarified the tradeoff between aerial geometry and active gain under explicit detection and hardware restrictions.

## Figures and Tables

**Table 1:** Baseline simulation parameters.

Parameter	Symbol	Value
Alice location	$q_{A}$	(0,0,0) m
Bob location	$q_{B}$	(800,600,0) m
Willie location	$q_{W}$	(500,200,0) m
Willie uncertainty radius	$r_{W}$	0 m in the baseline
UAV altitude	*H*	100 m
IRS element count	*M*	16
Alice transmit power	$P_{A}$	30 dBm
Jamming power	$P_{J}$	20 dBm
IRS output power budget	$P_{\max}$	30 dBm
Bob and Willie noise powers	$\sigma_{B}^{2},\sigma_{W}^{2}$	−80 dBm
IRS circuit-noise power	$\sigma_{U}^{2}$	−70 dBm
Reference gain	$\beta_{0}$	−30 dB
Path-loss exponents	$\alpha_{L},\alpha_{\mathrm{NL}}$	2.2,3.5
Environment parameters	a,b	10,0.6
Covertness tolerance	ϵ	0.05
Observation length	*n*	1000
Maximum covert-outage probability	$p_{\mathrm{out}}^{\max}$	0.05
Direct-link path loss	$L_{AW}$	105–150 dB in the sensitivity test
Horizontal search region	Q	[−200,1000]×[−200,800] m^2^
Grid spacing	$\Delta_{x},\Delta_{y}$	10 m

## Data Availability

The original contributions presented in this study are included in the article. Further inquiries can be directed to the corresponding author.
